# Sociodemographic Factors Associated With HIV/HCV High-Risk Behaviors Among People Who Use Drugs on Methadone Maintenance Treatment: A 10-Year Observational Study

**DOI:** 10.3389/fpsyt.2021.707257

**Published:** 2021-09-14

**Authors:** Cong Liu, Yi-lei Ma, Xue-han Liu, Yan-ran Duan, Pu-lin Liu, Xia Wang, Ping Yin

**Affiliations:** ^1^Department of Epidemiology and Biostatistics, School of Public Health, Tongji Medical College, Huazhong University of Science and Technology, Wuhan, China; ^2^Wuhan Centers for Disease Control and Prevention, Wuhan, China

**Keywords:** people who use drugs, high-risk behaviors, HIV, HCV, sociodemography

## Abstract

**Background:** Sociodemographic factors have an impact worldwide on the behavior of people who use drugs (PWUD). This study attempts to clarify the sociodemographic factors related to HIV/HCV high-risk behaviors (injection drug use, syringe sharing, and multiple sex partners) among PWUD on methadone maintenance treatment (MMT) in the long term.

**Methods:** The 13,300 PWUD recruited into the MMT program were followed during 2006–2015. Generalized estimating equations were used to examine the relationship between sociodemographic characteristics and HIV/HCV high-risk behaviors.

**Results:** We found that male (vs. female), living alone (vs. living with family or relatives), temporary income, financial support from family/friends, and financial support from social welfare (vs. regular salary) were positively associated with injection drug use. Age of initial drug use was negatively associated with injection drug use and syringe sharing. For both genders, being unmarried (vs. married or in cohabitation), living with friends, living alone (vs. living with family or relatives), temporary income, financial supports from family/friends (vs. regular salary), being employed (vs. unemployed/between jobs) was positively associated. In contrast, age at baseline was negatively associated with having multiple sexual partners for both genders. Ethnic of non-Han (vs. Han) was positively associated with having multiple sexual partners simply for males. Being divorced or widowed (vs. married or cohabitated) was positively associated with having multiple sexual partners merely for females.

**Conclusion:** HIV/HCV high-risk behaviors correlated with certain sociodemographic factors of PWUD receiving MMT. There is a need for improving the well-being, employment, and housing status of PWUD on MMT to reduce their HIV/HCV risk behaviors.

## Introduction

Drug abuse is a critical public health problem worldwide. According to the World Drug Report 2020, an estimated 269 million people (5.3% of the global population aged 15–64) had used drugs, with 58 million people in the world had used opioids in 2018. There were ~11.3 million people who injected drugs worldwide, of which more than one million were infected with HIV and 5.5 million suffered from hepatitis C (1). There were 2.14 million drug users in China by the end of 2019, accounting for 0.16% of the total population, of which 807,000 (around 37.5%) were heroin users (2, 3). A study using a calibrated model based on Monte Carlo simulations estimated those entire drug users increased from 0.86 million to more than 3.12 million from 2000 to 2030 (4). According to Chinese national sentinel surveillance, the prevalence of HIV among drug users is ranged from 3.0 to 4.5%, and the HCV prevalence rate is 33.4–41.8% during 2010–2015 (5). A modeling study estimated that HIV and HCV prevalence expected to increase, respectively, from 10.9 and 61.7 to 19.0 and 69.1% among people using polydrugs (using synthetic drugs and heroin concurrently) from 2005 to 2035 (6).

High-risk behaviors for HIV and HCV infection include injection drug use (IDU), multi-person use of syringes, and having multiple sexual partners (MSP), which have been increasing in recent years (7–9). It is worth noting that a considerable proportion of PWUDs had been exposed to these high-risk behaviors in China. A literature review showed that the proportion of drug injection among heroin-only users remained at 82.2% during 2006–2015, while the ratio increased from 58.6 to 65.2% among people using polydrug during 2012–2015. The proportion of syringe sharing in people using polydrug remained stable in a range of 20–30% and decreased from 38.6 to 22.1% during 2005–2015 (6). The mean number of sexual partners in the heroin-only users and polydrug users was 1.04 and 5.3, respectively (6), 37% of male injecting drug users reported having MSP (10), and even 75.21% of methamphetamine users reported having sex with multiple partners after using drugs (11). Therefore, the high-risk behaviors of PWUD and the resulting HIV/HCV infection and transmission have become a paramount public health concern in China.

Methadone Maintenance Treatment (MMT) programs were introduced in China in 2004. The primary goal of these programs is reducing opioid dependence, thereby contributing to HIV/HCV prevention and harm reduction. Several studies have shown that MMT programs successfully prevent HIV/HCV transmission and reduce high-risk behaviors among PWUD in China (12–14). A retrospective study showed that MMT clients' HIV and HCV seroconversion rates decreased significantly over a 7-year follow-up (12). Drug injections, syringe sharing, and commercial sex behavior significantly reduced at 12 months following MMT initiation (13, 14). However, we found that in our study population, that is, among PWUD treated with methadone, there were still a considerable number of high-risk behaviors for HIV and HCV infection. Such as injection drug use, sharing syringes, having MSP, and unprotected commercial sex. In addition, we should not ignore is that many studies on PWUD have shown sociodemography was closely related to high-risk behaviors for HIV and HCV infection. Several studies demonstrated that the younger the individual, the higher the risk of IDU (14–16), needle sharing (14, 17, 18), and having MSP (14, 19). Still, other studies showed that age was positively correlated with IDU (20, 21). Controversial opinions on gender (14, 19, 22), education (14, 16, 23), and marital status (14, 16, 24) had been raised. Earlier studies also showed that lower socioeconomic status (25) was positively correlated with IDU, unstable incomes (26) were positively correlated with syringe sharing. Homelessness (15, 24) and unemployment (16, 27) were positively correlated with injecting drug use, syringe sharing, unprotected sex (24), and having MSP (28). Besides, people who injected drugs were more likely to live with their parents (29).

Given that sociodemographic characteristics significantly impact HIV/HCV high-risk behaviors, it is necessary to explore whether high-risk behaviors among PWUD on MMT are related to specific demographic factors. Moreover, this will help us understand the factors influencing high-risk behaviors of people receiving MMT and enable health providers to implement targeted behavioral interventions. Previous studies on the associations between sociodemography and high-risk behavior rarely involved patients receiving MMT, and most of them were small sample and cross-sectional studies. Therefore, it is crucial to clarify the evidence of large-scale and longitudinal studies to study the associations among PWUD on MMT.

Wuhan is the largest city in China with an MMT program. The program has been active since early 2006, with 16 MMT clinics currently providing services for PWUD. Although more than 10 years of experience providing opioid dependence therapy, few studies have shown the relationship between sociodemographic factors and HIV/HCV high-risk behavior in PWUD on MMT. Based on a better understanding of the impact of social demographics on high-risk behaviors in this population, health providers can provide more tailored and effective treatment in Wuhan.

In this study, we first described the sociodemographic characteristics of PWUD on MMT and three high-risk behaviors associated with HIV/HCV infection from 2006 to 2015. Then, we constructed the generalized estimation equations based on these data to explore the factors affecting high-risk behaviors.

## Methods

### Design

A retrospective observational study was implemented among the PWUD on MMT by 10 years (2006–2015). The baseline investigation was conducted at the time of MMT enrollment, and the first follow-up visit was carried out within 6 months of enrollment. Then, yearly follow-up visits were conducted. Participants were followed longitudinally and completed between 1 and 10 follow-ups. All baseline and follow-up surveys were conducted under The Guidelines of AIDS Prevention in PWUD at MMT Clinics by the Chinese Center for Disease Control and Prevention (30).

### Setting and Participants

All participants are PWUD who applied voluntarily and gave informed consent for treatment, baseline, and follow-up between 2006 and 2015 at any of the MMT clinics in Wuhan, China. The eligibility criteria for MMT are as follows: (1) 18 years or more; (2) full capacity for civil conduct; (3) history of opioid use by any route of intake; (4) urine sample positive for opioid use before enrollment. The informed consent was formulated according to The Guidelines of AIDS Prevention in PWUD at MMT Clinics (30).

### Data Collection

Data for this observational study came from the National MMT Data Management System (NMDMS), established in 2005 and officially operated at Wuhan MMT Clinics in January 2006. The NMDMS contains information on outpatient management, staff status, patient treatment, and drug inventory. Among them, patient treatment information includes informed consent, enrollment application, medical examination, urine test records, treatment status, and baseline and follow-up survey data that we were authorized to obtain. Our research captured data records between January 1, 2006, and December 31, 2015, from the NMDMS, following the inclusion criteria: PWUD enrolled in the MMT during 2006–2015 and completed the baseline survey at the enrollment.

The generation of NMDMS data was required to comply with The Guidelines of AIDS Prevention in PWUD at MMT Clinics (30). The baseline and follow-up questionnaires were conducted in a private space by clinic staff through face-to-face interviews. Before the formal interview, the Chinese Center for Disease Control and Prevention trained these staff and conducted mock interviews to improve their investigation skills.

### Variables and Measurements

There were mainly two types of variables: sociodemography and HIV/HCV high-risk behaviors measured in our study. Social demographic variables include age, gender, ethnicity, education, marital status, living situation, income source, employments, age of initial drug use, and sexual activity in the last 3 months. High-risk behavioral variables include intravenous drug use in the last month, syringe sharing in the last month, and multiple sexual partners in the last 3 months.

Age, gender, ethnicity, and age of first drug use were only measured at baseline, and other variables were measured at baseline and all follow-up visits.

The Guidelines of AIDS Prevention in PWUD at MMT Clinics (30) defined the variables of the age of initial drug use, injection in the last month, syringe sharing in the last month, having multiple sex partners in the last 3 months.

Age of initial drug use was defined as the age of first use of opioids by injection, oral, and all other routes of opioid intake.

Injection in the last month was defined as used drugs intravenously at least once within the last month.

Syringe sharing in the last month was defined as sharing needles with others at least once among people who injection within the last month.

Having multiple sex partners in the last 3 months was defined as having more than one sex partner within the last 3 months among all participants on MMT who had sex in the last 3 months.

### Statistical Analysis

We summarized the baseline sociodemographic variables and HIV/HCV high-risk behaviors using mean and standard deviation for continuous variables and percentages for categorical variables.

Since we recorded repeated measurements for each participant, we used a generalized estimating equation (GEE) for binary outcomes and a logit link function to evaluate the independent association of social demographics with HIV/HCV high-risk behaviors throughout the 10-year follow-up period (31). Next, the autocorrelation structure was selected as the working correlation matrix, and all social-demographic variables were entered into a fixed multivariable logistic model.

All analyses were performed using IBM SPSS19.0. For all statistical tests, 2-sided, *p* < 0.05 were considered statistically significant. Missing observations (<5%) were excluded when SPSS executes statistical procedures.

### Ethics Approval

The study was approved by the Institutional Review Board of Wuhan Centers for Disease Control & Prevention in 2016 and has been performed under the ethical standards laid down in the 1964 Declaration of Helsinki and its later amendments.

### Data Statement

The data that support the findings of this study are available from the national MMT data management system. Still, restrictions apply to the availability of these data, which were used under license for the current research, and so are not publicly available. However, data are available from the authors upon reasonable request and with the permission of the Chinese Center for Disease Control and Prevention.

## Results

The social demography and HIV/HCV high-risk behaviors of participants were shown in Table 1. Between 2006 and 2015, a total of 13,300 PWUD participated in the Wuhan MMT program. The number of participants gradually declined after the baseline survey. We finally followed these patients through combined 54,300 visits for a median number of 3 follow-up visits, with the drop-out rate being 9.89% per year. A few participants were no-reply for behaviors, such as 222 (1.7%), 354 (2.7%), and 384 (5.6%) participants declined, respectively, to respond for injection drug use, sexual activity, and multiple sexual partners (MSP) at the survey of baseline.

**Table 1:** Social-demography and HIV/HCV high-risk behaviors of 13,300 PWUD on MMT during 2006–2015, Wuhan, China (mean/n, sd/percentage).

		**Baseline**	**Follow-up**									
			**1**	**2**	**3**	**4**	**5**	**6**	**7**	**8**	**9**	**10**
Age of initial drug use	Mean	28.16	28.17	28.16	28.14	28.10	28.02	27.88	27.70	27.36	26.57	25.92
	Sd	7.518	7.526	7.503	7.545	7.597	7.560	7.347	7.160	6.979	6.561	5.903
	*(n)*	13,083	12,871	8,715	6,192	4,460	3,191	2,207	1,467	881	382	144
Gender	Male	9,705	9,555	6,457	4,579	3,323	2,384	1,669	1,127	665	291	109
		73.0%	73.0%	73.1%	73.1%	73.9%	74.1%	75.1%	76.4%	75.1%	75.8%	75.2%
	Female	3,595	3,533	2,372	1,681	1,173	833	552	348	220	93	36
		27.0%	27.0%	26.9%	26.9%	26.1%	25.9%	24.9%	23.6%	24.9%	24.2%	24.8%
	*(sum.)*	13,300	13,088	8,829	6,260	4,496	3,217	2,221	1,475	885	384	145
Ethnic	Han	13,204	12,994	8,768	6,217	4,466	3,199	2,208	1,465	881	381	144
		99.3%	99.3%	99.3%	99.3%	99.3%	99.4%	99.4%	99.3%	99.5%	99.2%	99.3%
	Non-Han	96	94	61	43	30	18	13	10	4	3	1
		0.7%	0.7%	0.7%	0.7%	0.7%	0.6%	0.6%	0.7%	0.5%	0.8%	0.7%
	*(sum.)*	13,300	13,088	8,829	6,260	4,496	3,217	2,221	1,475	885	384	145
Education	Under junior middle school	901	886	579	394	280	191	140	96	51	19	8
		6.8%	6.8%	6.6%	6.3%	6.2%	5.9%	6.3%	6.5%	5.8%	4.9%	5.5%
	Junior middle school	8,805	8,650	5,740	4,073	2,882	2,067	1,429	947	562	244	94
		66.2%	66.1%	65.0%	65.1%	64.1%	64.3%	64.3%	64.2%	63.5%	63.5%	64.8%
	Senior High School or above	3,594	3,552	2,510	1,793	1,334	959	652	432	272	121	43
		27.0%	27.1%	28.4%	28.6%	29.7%	29.8%	29.4%	29.3%	30.7%	31.5%	29.7%
	*(sum.)*	13,300	13,088	8,829	6,260	4,496	3,217	2,221	1,475	885	384	145
Married status	Unmarried	5,078	4,986	3,297	2,288	1,626	1,162	800	553	352	158	55
		38.2%	38.1%	37.3%	36.5%	36.2%	36.1%	36.0%	37.5%	39.8%	41.1%	37.9%
	Divorced or widowed	2,361	2,331	1,605	1,164	862	595	384	262	148	70	24
		17.8%	17.8%	18.2%	18.6%	19.2%	18.5%	17.3%	17.8%	16.7%	18.2%	16.6%
	Married or in cohabitation	5,861	5,771	3,927	2,808	2,008	1,460	1,037	660	385	156	66
		44.1%	44.1%	44.5%	44.9%	44.7%	45.4%	46.7%	44.7%	43.5%	40.6%	45.5%
	*(sum.)*	13,300	13,088	8,829	6,260	4,496	3,217	2,221	1,475	885	384	145
Inhabitation	Solo living	1,539	1,252	600	343	204	142	123	59	33	8	6
		11.6%	10.3%	6.9%	5.5%	4.6%	4.4%	5.5%	4.0%	3.7%	2.1%	4.2%
	Live with friends	1,514	1,591	875	501	296	164	97	74	47	25	14
		11.4%	13.0%	10.1%	8.1%	6.6%	5.1%	4.4%	5.0%	5.3%	6.6%	9.7%
	Live with family or relatives	10,247	9,364	7,159	5,370	3,982	2,899	1,997	1,340	804	347	124
		77.0%	76.7%	82.9%	86.4%	88.8%	90.5%	90.1%	91.0%	91.0%	91.3%	86.1%
	*(sum.)*	13,300	12,207	8,634	6,214	4,482	3,205	2,217	1,473	884	380	144
Income	Social welfare	272	169	106	72	45	52	29	18	7	1	0
		2.0%	1.4%	1.2%	1.2%	1.0%	1.6%	1.3%	1.2%	0.8%	0.3%	0.0%
	Support by family or friends	7,827	5,556	3,769	2,380	1,526	1,139	733	400	197	47	11
		58.8%	45.4%	43.7%	38.2%	34.0%	35.5%	33.0%	27.2%	22.4%	12.3%	7.8%
	Temporary wage	3,715	4,471	3,057	2,168	1,635	1,016	875	631	423	216	81
		27.9%	36.6%	35.4%	34.8%	36.5%	31.7%	39.4%	42.8%	48.1%	56.5%	57.4%
	Regular salary	1,486	2,030	1,697	1,605	1,279	1,001	582	424	253	118	49
		11.2%	16.6%	19.7%	25.8%	28.5%	31.2%	26.2%	28.8%	28.8%	30.9%	34.8%
	*(sum.)*	13,300	12,226	8,629	6,225	4,485	3,208	2,219	1,473	880	382	141
Employment	Unemployed/between jobs	9,933	7,654	5,030	3,477	2,322	1,725	1,116	705	449	170	63
		74.7%	62.5%	58.5%	56.3%	52.1%	54.0%	50.5%	47.9%	50.8%	44.4%	43.4%
	Employed	3,367	4,587	3,563	2,704	2,135	1,468	1,093	767	435	213	82
		25.3%	37.5%	41.5%	43.7%	47.9%	46.0%	49.5%	52.1%	49.2%	55.6%	56.6%
	*(sum.)*	13,300	12,241	8,593	6,181	4,457	3,193	2,209	1,472	884	383	145
Injection in last month^*^	No	4,375	9,733	7,258	5,442	3,956	2,758	1,982	1,386	838	356	134
		33.5%	79.5%	84.3%	88.0%	88.8%	86.3%	89.7%	94.2%	95.2%	93.2%	93.1%
	Yes	8,703	2,515	1,351	739	501	436	227	85	42	26	10
		66.5%	20.5%	15.7%	12.0%	11.2%	13.7%	10.3%	5.8%	4.8%	6.8%	6.9%
	*(sum.)*	13,078	12,248	8,609	6,181	4,457	3,194	2,209	1,471	880	382	144
Syringe sharing in last month^†^	No	8,154	2,339	1,260	682	443	396	176	77	37	25	10
		93.7%	93.0%	93.3%	92.3%	88.4%	90.8%	77.5%	90.6%	88.1%	96.2%	100.0%
	Yes	549	177	91	57	58	40	51	8	5	1	0
		6.3%	7.0%	6.7%	7.7%	11.6%	9.2%	22.5%	9.4%	11.9%	3.8%	0.0%
	*(sum.)*	8,703	2,516	1,351	739	501	436	227	85	42	26	10
Had sex in last 3 months^*^	No	6,069	4,808	3,169	1,422	722	323	229	192	115	73	45
		46.9%	39.3%	36.6%	22.8%	16.1%	10.0%	10.3%	13.0%	13.0%	19.1%	31.0%
	Yes	6,877	7,418	5,485	4,818	3,770	2,892	1,991	1,282	767	309	100
		53.1%	60.7%	63.4%	77.2%	83.9%	90.0%	89.7%	87.0%	87.0%	80.9%	69.0%
	*(sum.)*	12,946	1,2226	8,654	6,240	4,492	3,215	2,220	1,474	882	382	145
Multiple sexual partners in last 3 months^‡^	No	5,817	6,963	5,338	4,714	3,702	2,858	1,975	1,271	766	308	99
		89.6%	95.3%	97.9%	98.4%	99.0%	99.4%	99.6%	99.7%	100.0%	100.0%	99.0%
	Yes	676	345	115	75	36	17	8	4	0	0	1
		10.4%	4.7%	2.1%	1.6%	1.0%	0.6%	0.4%	0.3%	0.0%	0.0%	1.0%
	*(sum.)*	6,493	7,308	5,453	4,789	3,738	2,875	1,983	1,275	766	308	100

* *The denominator of the percentage is all participants of PWUD on MMT.*

† *The denominator of the percentage is participants who had been injecting drugs in the last month.*

‡ *The denominator of the percentage is participants who had sex in the last 3 months*.

The majority of participants are male (73.0%), of Han ethnicity (99.3%), educated through junior year of high school (66.2%), married or living with others (44.1%), unemployed or between jobs (74.7%), supported by family or friends (58.8%), and living with family or relatives (77.0%). The baseline means age is 39.30 years (SD: 7.419), and the mean age of initial drug use is 28.16 years (SD: 7.518). At baseline, 8,703 (66.5%) and 549 (6.3%) participants, respectively, reported IDU and needle sharing within the past month. 6,877 (53.1%) participants said they had sexual activity in the last 3 months, and 676 (10.4%) of them (6,493 responded) reported having multiple sexual partners during the periods.

The results of multivariable GEE analyses with a summary of associations with IDU and syringe sharing were given, respectively, in Tables 2, 3. In multivariable GEE analyses, age of initial drug use [Adjusted Odds Ratio (AOR): 0.984, 95% Confidence Interval (CI): 0.979–0.988], gender (AOR: 0.936, 95% CI: 0.889–0.985), living alone (AOR: 1.545, 95% CI: 1.425–1.674), temporary income source (AOR: 1.137, 95% CI: 1.062–1.218), financial support from friends or family (AOR: 2.840, 95% CI: 2.651–3.043), and financial support from social welfare (AOR: 2.154, 95% CI: 1.808–2.567) remained independently associated with IDU within the past month. Age of initial drug use (AOR: 0.982, 95% CI: 0.968–0.996) remained independently associated with syringe sharing within the past month.

**Table 2:** Univariable GEE analyses of factors associated with injection and syringe sharing among PWUD on MMT in Wuhan, China, 2006–2015.

	**Injection^**†**^**	**Syringe sharing^**†**^**
Age at baseline		
Per year older	0.995 (0.992–0.998)^*^	0.981 (0.972–0.990)^*^
Age of initial drug use		
Per year older	0.987 (0.984–0.990)^*^	0.978 (0.968–0.987)^*^
Gender		
Male	Reference	Reference
Female	1.004 (0.956–1.053)	1.054 (0.907–1.225)
Ethnic		
Non-Han	Reference	Reference
Han	1.101 (0.851–1.425)	0.801 (0.391–1.639)
Education		
Senior high school or above	Reference	Reference
Junior middle school	1.094 (1.041–1.149)^*^	1.089 (0.932–1.273)
Under junior middle school	1.021 (0.929–1.122)	0.948 (0.700–1.284)
Married status		
Married or in cohabitation	Reference	Reference
Divorced or widowed	1.138 (1.072–1.209)^*^	1.134 (0.940–1.367)
Unmarried	1.121 (1.069–1.175)^*^	1.224 (1.056–1.419)^*^
Living situation		
Live with family or relatives	Reference	Reference
Live with friends	0.906 (0.847–0.970)^*^	0.880 (0.698–1.110)
Living alone	1.458 (1.359–1.564)^*^	1.008 (0.816–1.244)
Income source		
Regular salary	Reference	Reference
Temporary wage	1.180 (1.104–1.262)^*^	0.977 (0.773–1.235)
Support by family or friends	2.930 (2.752–3.120)^*^	0.951 (0.772–1.171)
Social welfare	2.475 (2.088–2.933)^*^	0.967 (0.575–1.629)
Employment		
Employed	Reference	Reference
Unemployed/between jobs	1.775 (1.699–1.854)^*^	0.900 (0.781–1.038)

† *In the last month.*

* *p < 0.05*.

**Table 3:** Multivariable GEE analyses of factors associated with injection and syringe sharing among drug users on MMT in Wuhan, China, 2006–2015.

	**Injection^**†**^**	**Syringe sharing^**†**^**
Age at baseline		
Per year older	1.004 (0.999–1.009)	0.994 (0.980–1.008)
Age of initial drug use		
Per year older	0.984 (0.979–0.988)^*^	0.982 (0.968–0.996)^*^
Gender		
Male	Reference	Reference
Female	0.936 (0.889–0.985)^*^	1.017 (0.870–1.190)
Ethnic		
Non-Han	Reference	Reference
Han	0.948 (0.743–1.209)	0.799 (0.386–1.655)
Education		
Senior high school or above	Reference	Reference
Junior middle school	1.036 (0.985–1.091)	1.099 (0.940–1.285)
Under junior middle school	0.937 (0.850–1.032)	0.944 (0.695–1.282)
Married status		
Married or in cohabitation	Reference	Reference
Divorced or widowed	1.043 (0.979–1.111)	1.089 (0.920–1.289)
Unmarried	0.995 (0.943–1.051)	1.192 (0.987–1.440)
Living situation		
Live with family or relatives	Reference	Reference
Live with friends	1.074 (1.000–1.154)	0.868 (0.686–1.099)
Living alone	1.545 (1.425–1.674)^*^	0.989 (0.792–1.235)
Income source		
Regular salary	Reference	Reference
Temporary wage	1.137 (1.062–1.218)^*^	0.981 (0.772–1.247)
Support by family or friends	2.840 (2.651–3.043)^*^	1.022 (0.807–1.293)
Social welfare	2.154 (1.808–2.567)^*^	1.117 (0.654–1.910)
Employment		
Employed	Reference	Reference
Unemployed/between jobs	1.039 (0.986–1.094)	0.859 (0.725–1.017)

† *In the last month.*

* *p < 0.05*.

The results obtained from the GEE analyses of association with multiple sexual partners (MSP) according to gender were presented in Tables 4, 5. For males, age at baseline (AOR: 0.968, 95% CI: 0.953–0.983), ethnic (AOR: 0.412, 95% CI: 0.249–0.683), being unmarried (AOR: 1.386, 95% CI: 1.152–1.667), living with friends (AOR: 2.885, 95% CI: 2.331–3.572), living alone (AOR: 4.205, 95% CI: 3.460–5.110), temporary income source (AOR: 1.734, 95% CI: 1.392–2.159), financial support from friends or family (AOR: 2.875, 95% CI: 2.305–3.586), and employment (AOR: 0.527, 95% CI: 0.451–0.616) were independently correlated with having MSP within the past 3 months. While for females, age at baseline (AOR: 0.956, 95% CI: 0.926–0.986), being divorced or widowed (AOR: 1.896, 95% CI: 1.295–2.776), being unmarried (AOR: 1.860, 95% CI: 1.335–2.593), living with friends (AOR: 3.159, 95% CI: 2.287–4.364), living alone (AOR: 3.962, 95% CI: 2.740–5.731), temporary income source (AOR: 1.566, 95% CI: 1.065–2.303), financial support from friends or family (AOR: 2.302, 95% CI: 1.573–3.369), and employment (AOR: 0.659, 95% CI: 0.511–0.850) remained independently associated with having MSP within the past 3 months.

**Table 4:** Univariable GEE analyses of factors associated with multiple sex partners in the last 3 months among PWUD on MMT in Wuhan, China, 2006–2015.

	**Male**	**Female**
Age at baseline		
Per year older	0.960 (0.951–0.970)^*^	0.939 (0.922–0.957)^*^
Age of initial drug use		
Per year older	0.967 (0.958–0.977)^*^	0.956 (0.937–0.975)^*^
Ethnic		
Non-han	Reference	Reference
Han	0.372 (0.221–0.627)^*^	^†^
Education		
Senior high school or above	Reference	Reference
Junior middle school	1.006 (0.850–1.191)	1.007 (0.762–1.332)
Under junior middle school	0.972 (0.711–1.328)	0.565 (0.281–1.140)
Married status		
Married or in cohabitation	Reference	Reference
Divorced or widowed	1.398 (1.129–1.730)^*^	2.159 (1.514–3.078)^*^
Unmarried	1.918 (1.629–2.260)^*^	3.155 (2.396–4.155)^*^
Living situation		
Live with family or relatives	Reference	Reference
Live with friends	2.833 (2.330–3.444)^*^	3.679 (2.731–4.956)^*^
Living alone	4.303 (3.588–5.160)^*^	4.858 (3.481–6.778)^*^
Income source		
Regular salary	Reference	Reference
Temporary wage	2.083 (1.683–2.579)^*^	2.219 (1.559–3.160)^*^
Support by family or friends	2.074 (1.683–2.555)^*^	2.132 (1.506–3.018)^*^
Social welfare	1.618 (0.850–3.078)	3.455 (0.940–12.702)
Employment		
Employed	Reference	Reference
Unemployed/between jobs	0.823 (0.722–0.938)^*^	0.949 (0.764–1.179)

† *Due to the small sample size of non-Han women during the follow-up, ethnic variables were not included.*

* *p < 0.05*.

**Table 5:** Multivariable GEE analyses of factors associated with multiple sex partners in the last 3 months among PWUD on MMT in Wuhan, China, 2006–2015.

	**Male**	**Female**
Age at baseline		
Per year older	0.968 (0.953–0.983)^*^	0.956 (0.926–0.986)^*^
Age of initial drug use		
Per year older	0.997 (0.983–1.012)	1.003 (0.974–1.033)
Ethnic		
Non-Han	Reference	Reference
Han	0.412 (0.249–0.683)^*^	^†^
Education		
Senior high school or above	Reference	Reference
Junior middle school	0.965 (0.812–1.146)	0.983 (0.736–1.312)
Under junior middle school	0.872 (0.631–1.206)	0.557 (0.275–1.130)
Married status		
Married or in cohabitation	Reference	Reference
Divorced or widowed	1.213 (0.968–1.520)	1.896 (1.295–2.776)^*^
Unmarried	1.386 (1.152–1.667)^*^	1.860 (1.335–2.593)^*^
Living situation		
Live with family or relatives	Reference	Reference
Live with friends	2.885 (2.331–3.572)^*^	3.159 (2.287–4.364)^*^
Living alone	4.205 (3.460–5.110)^*^	3.962 (2.740–5.731)^*^
Income source		
Regular salary	Reference	Reference
Temporary wage	1.734 (1.392–2.159)^*^	1.566 (1.065–2.303)^*^
Support by family or friends	2.875 (2.305–3.586)^*^	2.302 (1.573–3.369)^*^
Social welfare	1.330 (0.659–2.686)	3.126 (0.735–13.305)
Employment		
Employed	Reference	Reference
Unemployed/between jobs	0.527 (0.451–0.616)^*^	0.659 (0.511–0.850)^*^

† *Due to the small sample size of non-Han women during the follow-up, ethnic variables were not included.*

* *p < 0.05*.

## Discussion

Our data revealed several sociodemographic factors related to HIV/HCV high-risk behaviors in PWUD on MMT. Temporary income source, financial support from family or friends, or social welfare (vs. regular salary), male (vs. female), living alone (vs. with family or relatives) had a positive association with IDU. And, older age at initial drug use negatively associated with IDU and syringe sharing within the past month. Besides, younger baseline age, unmarried (vs. married or in cohabitation), living with friend or living alone (vs. with family or relatives), temporary income source, financial support from family or friends (vs. regular salary), and being employed (vs. unemployed/between jobs) had a positive association with MSP for both genders. However, ethnic Han (vs. non-Han) had a negative association only for males, divorced or widowed (vs. married or in cohabitation) had a positive association with having MSP within the 3 months only for females.

The results of our study indicated that temporary income, financial assistance from family or friends, and social welfare assistance were positively associated with IDU within the past month. Two cross-sectional studies showed that most people who injected drugs relied on financial help from relatives or friends (29) and even obtained money illegally (32). Therefore, considering our study has a long-duration retrospective design, we can confidently say that income source is an influencing factor of injecting drugs. Consistent with the previous study (14), we also found that male PWUD were more likely to inject drugs than female PWUD. Moreover, our research further supported evidence from the earlier study, which showed that the older the age of initial drug use, the lower the risk of IDU (33). These findings suggested that local authorities could increase social welfare budgets and vocational training resources for PWUD to obtain reliable employment opportunities and income and strengthen early intervention on harm reduction of drug use for young people. We found that PWUD who lived alone was more likely to inject drugs. However, this finding was inconsistent with a prior study from Russia, which showed that people who injected drugs were more likely to live with their parents (29). Considering that the study samples came from two different countries, further research is needed to clarify the relationship between living situations and IDU.

Our study also showed that the age of initial drug use was negatively correlated with syringe sharing within the past month. Although two previous studies demonstrated that the older the age at initial IDU, the less likely people were to share syringes (34, 35), there was no research illustrating the direct relationship between age of initial drug use (whether IDU or not) and syringe sharing. Our findings provided direct evidence for the association between age and syringe sharing and suggested the importance of early intervention and health education on drug misuse for adolescents.

Another important finding was that living with friends, living alone, having a temporary income, and financial assistance from family or friends were positively associated with having MSP within the past 3 months for both genders. In previous literature, economic vulnerability (36), residential stability (37), and residential statuses (38) were shown to be the factors of having MSP among women and young people. However, there is limited research about the relationship between MSP and residential status or income source in PWUD. Our novel findings reveal critical information that helps to provide targeted social support for PWUD.

Previous studies have demonstrated that marital status, employment, and age were related to MSP among IV drug users (28, 39) or club drug users (11), and younger PWUD was more likely to report multiple recent unprotected sexual encounters with different partners (40). Consistent with these studies, we also found that the age at baseline was negatively associated while unmarried and employed were positively independently associated with having MSP within the past 3 months in PWUD on MMT. These results will help the health sector implement targeted behavioral intervention strategies.

We found divorced and widowed were positively correlated with MSP only for female PWUD while not for male PWUD. A possible explanation is that traditional Chinese culture requires women to strictly abide by marital ethics (while men are much more relaxed), resulting in the proportion of extramarital sex being lower among Chinese women than in men (41, 42). In contrast, it is plausible that women are less likely to be restricted by marital ethics after divorce or widowhood, so their sexual behavior patterns, namely the number of sexual partners, have changed to more significant than men. Another interesting finding of this study was that ethnic Han was negatively correlated with MSP among male PWUD. That may be related to the fact that most non-Han ethnic minorities in Wuhan are immigrants, and the immigrant population's level of sexual risk behavior is relatively high (43, 44). Further work is required to enhance our understanding of the differences and the implications for health provision.

It is worth noting that as many as 47% of the PWUD at baseline did not have sex in the last 3 months, while up to 67% of the PWUD at baseline injected in the latest month. Hence in comparison to sex behavior risk, the injecting behavior risk continues to be much more critical for PWUD. This finding has significant implications for designing clinical and public health interventions, focusing on reducing intravenous behavior and controlling the sharing of syringes for PWUD on MMT. For example, it is necessary to enhance public education on the risks of intravenous injections and severe crackdowns on multiple injection sites.

There were several limitations to our study. First, our patient population—PWUD receiving MMT—is not a random sample of the drug-dependent population and therefore not generalizable for PWUD as a whole. Second, as a 10-year retrospective study, loss to follow-up is predictably an issue. Third, the data rely on self-reported information and are susceptible to recall bias, as well as socially influenced reporting (45). Fourth, several confounders outside of social demographics are not measured here. Such as other drug use/dependence (besides opioids, e.g., alcohol and stimulant), treatment-related variables (the dose of methadone, compliance, and regularity), and psychosocial factors could influence the association between variables. The results of HIV testing were also not considered in this study. Since people tend to change their behaviors after HIV/HCV testing is positive, the clinical and public health implications of high-risk behaviors in HIV-negative individuals are very different. Further studies, which take these variables into account, will need to be undertaken.

## Conclusions

The purpose of the current study was to clarify the sociodemographic factors related to HIV/HCV high-risk behaviors among PWUD on MMT in the long term. Our study has shown that several HIV/HCV high-risk behaviors independently associated with baseline age (for MSP), age at initial drug use (for IDU and syringe sharing), living situation (for IDU and MSP), income source (for IDU and MSP), marital status (for MSP), and employment status (for MSP). These findings hint that certain sociodemographic variables have long-term effects on high-risk behaviors of PWUD on MMT. Hence, there is a need for improving the well-being, employment, and housing status of PWUD on MMT to reduce their HIV/HCV risk behaviors. Despite its limitations, the study certainly adds to our understanding of the association between sociodemography and high-risk behaviors of PWUD.

## Data Availability Statement

The data that support the findings of this study are available from the national MMT data management system. Still, restrictions apply to the availability of these data, which were used under license for the current research, and so are not publicly available. However, data are available from the authors upon reasonable request and with the permission of the Chinese Center for Disease Control and Prevention. Requests to access the datasets should be directed to Cong Liu, liucong@whcdc.org.

## Ethics Statement

The study was approved by the Institutional Review Board of Wuhan Centers for Disease Control & Prevention and has been performed under the ethical standards laid down in the 1964 Declaration of Helsinki and its later amendments.

## Author Contributions

CL, XW, and PY performed the research. CL and PY designed the research study and wrote the protocol and conducted the statistical analysis. Y-lM, X-hL, Y-rD, and XW conducted literature searches and provided summaries of previous research studies. CL wrote the first draft of the manuscript. All authors contributed to and have approved the final manuscript.

## Funding

The study was supported by the National Natural Science Foundation of China (No. 81573262) and the Health and Family Planning Commission of Wuhan Municipality (No. WG17Q01). The funders had no role in study design, data collection and analysis, decision to publish, or preparation of the manuscript.

## Conflict of Interest

The authors declare that the research was conducted in the absence of any commercial or financial relationships that could be construed as a potential conflict of interest.

## Publisher's Note

All claims expressed in this article are solely those of the authors and do not necessarily represent those of their affiliated organizations, or those of the publisher, the editors and the reviewers. Any product that may be evaluated in this article, or claim that may be made by its manufacturer, is not guaranteed or endorsed by the publisher.

## References

[1] Niaz K Haar K Carpentier C Pietchman T. World Drug Report. (2020). Available online at: https://wdr.unodc.org/wdr2020/index2020.html (accessed August 25, 2021).

[2] Tang H Li M Yan X Lu Z Jia Z. Modeling the dynamics of drug spreading in China. Int J Environ Res Public Health. (2021) 18:288. 10.3390/ijerph18010288 33401693 PMC7796082

[3] Drug Situation in China. Office of China National Narcotics Control Committee. (2019). Available online at: http://www.nncc626.com/2020–06/25/c_1210675877.htm (accessed August 25, 2021).

[4] Su S Fairley CK Mao L Medland NA Jing J Cheng F. Estimates of the national trend of drugs use during 2000–2030 in China: a population-based mathematical model. Addict Behav. (2019) 93:65–71. 10.1016/j.addbeh.2019.01.022 30685570

[5] Lin GE Dongmin LI Peilong LI GUO-Wei Cui Y. Population specific sentinel surveillance for HIV infection, syphilis and HCV infection in China, during 2010–2015. Dis Surveill. (2017) 32:111–7. 10.3784/j.issn.1003-9961.2017.02.008

[6] Su S Fairley CK Mao L Medland N Shen M Li Y. Estimation of the impact of changing drug-use trend on HIV, hepatitis C and syphilis epidemics among people who use synthetic drug-only, polydrug and heroin-only during 2005–2035 in China: modelling study. Sex Transm Infect. (2020) 96:608–14. 10.1136/sextrans-2019-054360 32188771

[7] Ruta S Cernescu C. Injecting drug use: a vector for the introduction of new hepatitis C virus genotypes. World J Gastroenterol. (2015) 21:10811–23. 10.3748/wjg.v21.i38.10811 26478672 PMC4600582

[8] Wenz B Nielsen S Gassowski M Santoshövener C Cai W Ross RS. High variability of HIV and HCV seroprevalence and risk behaviours among people who inject drugs: results from a cross-sectional study using respondent-driven sampling in eight German cities (2011–14). BMC Public Health. (2016) 16:927. 10.1186/s12889-016-3545-4 27595567 PMC5011883

[9] Márványkövi F Melles K Rácz J. [Sex and drug: correlation of risk perception and behavioral patterns among intravenous drug users]. Psychiatria Hungarica. (2006) 21:241–55. 17090836

[10] Yao Y Wang N Chu J Ding G Jin X Sun Y. Sexual behavior and risks for HIV infection and transmission among male injecting drug users in Yunnan, China. Int J Infect Dis. (2009) 13:154–61. 10.1016/j.ijid.2008.05.1228 18778963 PMC2768780

[11] Bao YP Liu ZM Li JH Zhang RM Hao W Zhao M. Club drug use and associated high-risk sexual behaviour in six provinces in China. Addiction. (2015) 110:11–9. 10.1111/add.12770 25533860

[12] Zou X Ling L Zhang L. Trends and risk factors for HIV, HCV and syphilis seroconversion among drug users in a methadone maintenance treatment programme in China: a 7-year retrospective cohort study. BMJ Open. (2015) 5:e008162. 10.1136/bmjopen-2015-008162 26297365 PMC4550742

[13] Zhang L Chow EPF Zhuang X Liang YX Wang YF Tang CY. Methadone maintenance treatment participant retention and behavioural effectiveness in China: a systematic review and meta-analysis. PLoS ONE. (2013) 8:e68906. 10.1371/journal.pone.0068906 23922668 PMC3724877

[14] Wen C Xia Y Yan H Hall BJ Li L. Predictors of continued HIV-risk behaviors among drug users in methadone maintenance therapy program in China—a prospective study. Harm Reduct J. (2013) 10:23. 10.1186/1477-7517-10-23 24107380 PMC3853934

[15] DeBeck K Small W Wood E Li K Montaner J Kerr T. Public injecting among a cohort of injecting drug users in Vancouver, Canada. J Epidemiol Commun Health. (2009) 63:81–6. 10.1136/jech.2007.069013 18628270

[16] Cui SJ Li YQ Xiang YT Yan KJ Fan CX Chen Q. Demographic and clinical features of chinese heroin users who switch from non-injection to injection. Subst Use Misuse. (2015) 50:1739–46. 10.3109/10826084.2014.997826 26683897

[17] Kral AH Lorvick J Edlin BR. Sex- and drug-related risk among populations of younger and older injection drug users in adjacent neighborhoods in San Francisco. J Acquir Immune Defic Syndr. (2000) 24:162–7. 10.1097/00126334-200006010-00011 10935692

[18] Baldwin P Shrestha R Potrepka J Copenhaver M. The age of initiation of drug use and sexual behavior may influence subsequent HIV risk behavior: a systematic review. ISRN AIDS. (2013) 2013:976035. 10.1155/2013/976035 24381791 PMC3870609

[19] Somlai AM Kelly JA Benotsch E Gore-Felton C Ostrovski D McAuliffe T. Characteristics and predictors of HIV risk behaviors among injection-drug-using men and women in St. Petersburg, Russia. Aids Educ Prev. (2002) 14:295–305. 10.1521/aeap.14.5.295.23873 12212716

[20] Neupane SR Mishra SR Adhikari S Poudyal AK. Socio-Demographic correlates of injection drug use among male drug users: a cross sectional study in Nepal. J Commun Health. (2014) 39:1124–32. 10.1007/s10900-014-9867-1 24705679

[21] Kerr T Marshall BDL Miller C Shannon K Zhang R Montaner JSG. Injection drug use among street-involved youth in a Canadian setting. BMC Public Health. (2009) 9:171. 10.1186/1471-2458-9-171 19493353 PMC2697990

[22] Lei Z Di Z Wen C Xia Z Li L. High prevalence of HIV, HCV and tuberculosis and associated risk behaviours among new entrants of methadone maintenance treatment clinics in Guangdong Province, China. PLoS ONE. (2013) 8:e76931. 10.1371/journal.pone.0076931 24116185 PMC3792874

[23] White B Day C Degenhardt L Kinner S Fry C Bruno R. Prevalence of injecting drug use and associated risk behavior among regular ecstasy users in Australia. Drug Alcohol Depend. (2006) 83:210–7. 10.1016/j.drugalcdep.2005.11.014 16343810

[24] Neaigus A Reilly KH Jenness SM Hagan H Wendel T Gelpi-Acosta C. Dual HIV risk: receptive syringe sharing and unprotected sex among HIV-negative injection drug users in New York City. AIDS Behav. (2013) 17:2501–9. 10.1007/s10461-013-0496-y 23640654

[25] Amiri M Khosravi A Chaman R. Drug abuse pattern and high risk behaviors among addicts in Shahroud County of Semnan Province, Northeast Iran in 2009. J Res Health Sci. (2011) 10:254–63. 22911932

[26] DeBeck K Wood E Qi JZ Fu E McArthur D Montaner J. Interest in low-threshold employment among people who inject illicit drugs: implications for street disorder. Int J Drug Policy. (2011) 22:376–84. 10.1016/j.drugpo.2011.05.012 21684142 PMC3178688

[27] Reynolds GL Fisher DG Estrada AL Trotter R. Unemployment, drug use, and HIV risk among American Indian and Alaska native drug users. Am Indian Alsk Native Ment Health Res. (2000) 9:17–32. 10.5820/aian.0901.2000.17 11279551

[28] Todd CS Earhart KC Botros BA Khakimov MM Giyasova GM Bautista CT. Prevalence and correlates of risky sexual behaviors among injection drug users in Tashkent, Uzbekistan. AIDS Care. (2007) 19:122–9. 10.1080/09540120600852150 17129867

[29] Wall M Schmidt E Sarang A Atun R Renton A. Sex, drugs and economic behaviour in Russia: a study of socio-economic characteristics of high risk populations. Int J Drug Policy. (2011) 22:133–9. 10.1016/j.drugpo.2010.10.001 21055913

[30] The Guidelines of AIDS Prevention in PWUD at MMT Clinics. Beijing: Secretariat of the National Working Group of MMT, Chinese Center for Disease Control and Prevention (2004). p. 206.

[31] Lee JH Herzog TA Meade CD Webb MS Brandon TH. The use of GEE for analyzing longitudinal binomial data: a primer using data from a tobacco intervention. Addict Behav. (2007) 32:187–93. 10.1016/j.addbeh.2006.03.030 16650625

[32] Agha A Parviz S Younus M Fatmi Z. Socio-economic and demographic factors associated with injecting drug use among drug users in Karachi, Pakistan. J Pak Med Assoc. (2003) 53:511–6. 14738255

[33] Cheng Y Sherman SG Srirat N Vongchak T Kawichai S Jittiwutikarn J. Risk factors associated with injection initiation among drug users in Northern Thailand. Harm Reduct J. (2006) 3:10. 10.1186/1477-7517-3-10 16536869 PMC1450277

[34] Novelli LA Sherman SG Havens JR Strathdee SA Sapun M. Circumstances surrounding the first injection experience and their association with future syringe sharing behaviors in young urban injection drug users. Drug Alcohol Depend. (2005) 77:303–9. 10.1016/j.drugalcdep.2004.08.021 15734230

[35] Parviz S Fatmi Z Altaf A McCormick JB Fischer-Hoch S Rahbarc M. Background demographics and risk behaviors of injecting drug users in Karachi, Pakistan. Int J Infect Dis. (2006) 10:364–71. 10.1016/j.ijid.2005.07.010 16793307

[36] Dorina O Khangelani Z Nompumelelo Z Olive S Vuyelwa M. Determinants of multiple sexual partnerships in South Africa. J Public Health. (2015) 37:97–106. 10.1093/pubmed/fdu010 24639477

[37] German D Latkin CA. Social stability and HIV risk behavior: evaluating the role of accumulated vulnerability. AIDS Behav. (2012) 16:168–78. 10.1007/s10461-011-9882-5 21259043 PMC3163043

[38] Roy É Robert M Vaillancourt É Boivin JF Vandermeerschen J Martin I. Residential trajectory and HIV high-risk behaviors among Montréal Street Youth—a reciprocal relationship. J Urban Health. (2011) 88:767–78. 10.1007/s11524-011-9574-5 21494896 PMC3157499

[39] Assari S Yarmohamadivasel M Lankarani MM Sehat M Narenjiha H Rafiey H. Having multiple sexual partners among Iranian intra-venous drug users. Front Psychiatry. (2014) 5:125. 10.3389/fpsyt.2014.00125 25346698 PMC4193211

[40] Islam MM Topp L Conigrave KM Haber PS White A Day CA. Sexually transmitted infections, sexual risk behaviours and perceived barriers to safe sex among drug users. Aust N Z J Public Health. (2013) 37:311–5. 10.1111/1753-6405.12077 23895472

[41] Zhang N Parish WL Huang Y Pan S. Sexual infidelity in China: prevalence and gender-specific correlates. Arch Sex Behav. (2012) 41:861–73. 10.1007/s10508-012-9930-x 22544304

[42] Zheng W Zhou X Zhou C Liu W Li L Hesketh T. Detraditionalisation and attitudes to sex outside marriage in China. Cult Health Sex. (2011) 13:497–511. 10.1080/13691058.2011.563866 21452090

[43] Li X Zhang L Stanton B Fang X Xiong Q Lin D. HIV/AIDS-related sexual risk behaviors among rural residents in China: potential role of rural-to-urban migration. AIDS Educ Prev. (2007) 19:396–407. 10.1521/aeap.2007.19.5.396 17967110 PMC2064039

[44] Hong Y Stanton B Li X Yang H Lin D Fang X. Rural-to-urban migrants and the HIV epidemic in China. AIDS Behav. (2006) 10:421–30. 10.1007/s10461-005-9039-5 16421651 PMC1791012

[45] DeBeck K Kerr T Li K Milloy MJ Montaner J Wood E. Incarceration and drug use patterns among a cohort of injection drug users. Addiction. (2009) 104:69–76. 10.1111/j.1360-0443.2008.02387.x 19133890 PMC3731940

